# Statistical Evaluation and Optimization of Factors Affecting the Leaching Performance of Copper Flotation Waste

**DOI:** 10.1100/2012/758719

**Published:** 2012-05-02

**Authors:** Semra Çoruh, Sermin Elevli, Feza Geyikçi

**Affiliations:** ^1^Department of Environmental Engineering, Ondokuz Mayıs University, 55139 Samsun, Turkey; ^2^Department of Industrial Engineering, Ondokuz Mayıs University, 55139 Samsun, Turkey; ^3^Department of Chemical Engineering, Ondokuz Mayıs University, 55139 Samsun, Turkey

## Abstract

Copper flotation waste is an industrial by-product material produced from the process of manufacturing copper. The main concern with respect to landfilling of copper flotation waste is the release of elements (e.g., salts and heavy metals) when in contact with water, that is, leaching. Copper flotation waste generally contains a significant amount of Cu together with trace elements of other toxic metals, such as Zn, Co, and Pb. The release of heavy metals into the environment has resulted in a number of environmental problems. The aim of this study is to investigate the leaching characteristics of copper flotation waste by use of the Box-Behnken experimental design approach. In order to obtain the optimized condition of leachability, a second-order model was examined. The best leaching conditions achieved were as follows: pH = 9, stirring time = 5 min, and temperature = 41.5°C.

## 1. Introduction

Waste materials coming from municipal and industrial processes in Turkey are increasing, just as they are in many other countries. The release of large quantities of heavy metals into the natural environment due to hazardous waste leaching has resulted in a number of environmental problems [1, 2]. These waste materials may contain metals such as copper, lead, zinc, arsenic, cobalt, nickel, and chromium. For every tonne of blister copper production, about 2.2 tonnes of flotation waste are generated. In this regard, landfill disposal of copper flotation waste from copper flash smelting plant is not feasible since a few hundred tons are produced per year; leaching of heavy metals into groundwater is of concern [3]. The disposal of industrial waste materials is becoming more expensive each year due to large land areas needed for its disposal. The best way to solve the disposal problem of waste materials is to decrease the quantity for disposal with utilization of waste materials in the industry [4].

Copper is mined in open pits and below ground. The ore usually contains less than 1% copper and is often associated with sulfide minerals. One of two processing methods is used to refine concentrated copper. Pyrometallurgy, or smelting, is used on ore with copper sulfide and iron sulfide minerals. The concentrate is dried and fed into a furnace. The minerals are partially oxidized and melted, resulting in segregated layers. The matte layer refers to the iron-copper-sulfide mixture that sinks to the bottom. The slag, which refers to the remaining impurities, floats on top of the matte. The copper flotation waste (slag) generated from the copper industry is a by-product from the copper smelting industry [5–8]. The toxicity of the waste is determined by leaching tests and depending on the characteristics of the waste; the spent abrasive must be disposed of as solid or hazardous waste. Owing to the environmental impact of the process and waste management challenges faced for safe disposal of the wastes, the United States Environmental Protection Agency (US EPA) encourages industries, businesses, and institutions to consider all pollution prevention options to aid environmentally preferable purchasing (EPP). EPP will result in resource and energy conservation, waste minimization, and extension of landfill capacity [3, 9].

The Black Sea Copper Works is situated in the metropolitan city center of Samsun, Turkey. About 1.5–2 million tonnes of copper flotation waste arising from the factory are disposed of on the flood plain of the Yeşilırmak River, without any environmental pollution control [10]. The toxic metals present in the copper flotation waste, such as Cu, Zn, Co, and Pb, affect rich groundwater resources and surface waters by leaching due to rain water, which is very high in this region.

 The aim of the present study is to investigate whether or to what extent pH, stirring time, and temperature have an effect, either individually and/or jointly, on the leaching of copper flotation waste. The main effects and the interactions of these factors were studied at three levels using a Box-Behnken experimental design, which provides a second-order mathematical model, more information from fewer experiments, and optimum values of the experimental factors.

## 2. Materials and Experimental Procedure

### 2.1. Materials

Copper flotation waste has a black color and a glassy appearance. The specific gravity of copper flotation waste under investigation is 3100 kg/m^3^. The absorption capacity of the waste material is typically very low (0.13%). The chemical composition of the flotation waste was determined by using an X-ray fluorescence spectrometer (Rigaku, Rix-2000). The chemical composition of the waste, labeled as CFW in Table 1, shows iron oxides (67.68%) and silica (24.87%), together with some hazardous oxides, as ZnO, CuO, PbO, Cr_2_O_3_, and CoO. In previous studies, X-ray diffractometry (XRD) and scanning electron microscopy (SEM) spectra results show that copper flotation waste mainly consisted of magnetite (FeO·Fe_2_O_3_) and fayalite (2FeO·SiO_2_) [10].

### 2.2. Experimental Procedure

Leaching is a method to remove soluble components from a solid matrix. We can describe leaching by a very simple equation:

(1)   material  (leachee)+  leachant  →  leachate.

It can be assumed that the material to be leached is known, although its physical and chemical/mineralogical properties will affect the final result.

Leaching is one of the central unit operations in the hydrometallurgical processes. A recent survey of the literature identified more than 100 leaching methods to remove soluble components from a solid matrix. Several of these are regulatory methods, mandated to characterize materials; others are approved by organizations for establishing compliance to particular specifications. Many were developed for application to municipal solid waste or industrial wastes prior to use or disposal. Some are intended to mimic natural conditions, while the intent of others is to obtain information about the nature of the extractable material within a particular solid. The methods vary in the amount and particle size of the leached sample, the type and volume of leachant solution(s), and the leachant delivery method and time. Although some methods have been developed for specific types of materials, most leaching methods have been applied to a variety of materials [9, 11, 12].

Batch leaching studies were used to evaluate the leaching and pollution potentials of pollutants in copper flotation waste samples. Effects of the pH, stirring time, and temperature on leaching behaviors of the pollutants in the copper flotation waste were investigated in the batch leaching experiments. These experiments were carried out in the batch reactors containing liquid/solid (L/S) mass ratio of 10 (10 g solid for 100 mL of deionized water) and different pH, stirring time, and temperature. At the end of each experiment, the mixtures were filtered. The leachates were maintained to be highly acidic by adding nitric acid to prevent the metal ion precipitation. The samples were refrigerated at 4°C until analysis by atomic absorption spectrometry (AAS) was carried out. A UNICAM 929 model Atomic Absorption Spectrophotometer with air-acetylene flame was used for the determination of copper. The hallow cathode lamp for copper was operated at 6 mA and the wavelength was set at 327.4 nm using a slit-width of 1.0 nm. Detection limit of copper is 0.041 mg/L.

## 3. Experimental Design

In an experiment, one or more factors are deliberately changed in order to observe the effect of the changes on one or more response variables. When too many factors or factor levels need to be considered to produce sufficient data, there will be a huge number of repetitive experiments that need to be conducted. This would be prohibitive in practice from a time and cost viewpoint. Additionally, univariate methods do not take interactive effects between factors into account. Therefore, an efficient method of experimental planning is critical to produce credible data by concise experiment design.

The statistical design of experiments (DOE) is an efficient procedure for planning experiments so that the data obtained can be analyzed to yield valid and objective conclusions. The two main applications of experimental design are screening, in which the factors that influence the experiment are identified, and optimization, in which the optimal settings or conditions for an experiment can be found [13, 14]. The usual approach is to start with a screening design by including all possible experimental factors, to select the significant factors, to identify whether or not linearity assumption is violated, and then to continue with an experimental optimization design, such as Box-Behnken design.

Factorial experiments are experiments that investigate the effects of two or more factors or input parameters on the output response of a process. 2^*p*^  factorial experiment, where *p* is the number of factors, is a special case of factorial design and allows the experimenter to study several factors with two levels simultaneously.

The Box-Behnken design is an independent quadratic design in that it does not contain an embedded factorial or fractional factorial design; the treatment combinations are at the midpoints of edges of the process space and at the center.

### 3.1. Full Factorial Design

The metal mobility depends on the pH, stirring time, and temperature, as they influence the speciation and association of the metals in the waste material [15]. In order to obtain optimized conditions for the leachability of copper, a 2^3^ full factorial experimental design was used to evaluate the preliminary significance of the variables, as well as the interactions between them. The experimental variables, which are pH, stirring time, and temperature, were evaluated at two levels, low (denoted as −1) and high (denoted as +1), as shown in Table 2. The level selection for each factor was carried out on the basis of the preliminary trials and previous publishing results.

For  2^*k*^  factorial designs, it is assumed that the response is close to linear over the range of the factor levels. However, linearity assumption is often violated in practice. In this case, it is necessary to include one or more runs where all factors are set at their midpoint. The addition of center points to design allows the researcher to check whether the linearity of the effects is a reasonable assumption or whether quadratic terms should be added to the model. The results of the two-level statistical design for three-independent variables with two replicates and three center points (pH = 6, stirring time = 75.5 minutes, and temperature = 40°C) are shown in Table 3.

The data was processed using the Minitab 16 statistical computer program. The null hypothesis stating that the main effects, interactions, and the curvature equal to zero was tested by using *F*-test (Table 4). The small *P* values (<0.05) mean that not all the main effects and interactions are zero at the 5% significance level. In other words, there is reasonably strong evidence that at least some of the main effects and interactions are not equal to zero. Curvature term with *P* value lower than 0.05 indicates that there is curvature in the fitted data, and the response at the center point will be either higher or lower than the fitted value of the factorial (corner) points.

The estimated effects and coefficients are summarized in Table 5. It is clearly seen that the chosen experimental parameters are significant at the 5% level. Since experimental factors were found as significant in this step, they can be used in the optimization step. From examining the magnitude of the effects, pH is dominant, followed by the interaction of *BC* (stirring time and temperature). All the main and interaction effects, except pH, have positive effects on leachability. According to the coefficients, it can be concluded that one unit increase in pH will result in a 27.67 unit decrease in concentration of leached copper. The contribution of precipitation at the high pH levels causes this relationship. According to the determination coefficient (*R* ^2^-adj = 0.9016), which shows the explanatory power of model, only 9.84% of total variation was not explained.

### 3.2. Box-Behnken Design

2^3^ factorial design was used to identify whether or not the selected experimental factors are significant and whether or not curvature exists. Since curvature in fitted data was detected, the assumption of linear effects is not reasonable. Thus, a second-order model should be examined next, instead of a first-order model with interaction, in order to obtain an optimized condition of leachability.

Response surface methodology (RSM) is an experimental technique invented to find the optimal response within the specified ranges of the factors. These designs are capable of fitting a second-order prediction equation $\left(\hat{y}={\hat{\beta }}_{0}+\sum_{i=1}^{k}{\hat{\beta }}_{i}x_{i}+\sum_{i=1}^{k}{\hat{\beta }}_{ii}x_{i}^{2}+\sum \sum_{i<j}^{}{\hat{\beta }}_{ij}x_{i}x_{j}\right)$ for the response. The quadratic terms in these equations model the curvature in the true response function. The two most common designs generally used in response surface modeling are central composite and Box-Behnken designs (BBDs). In this study, BBD has been used for the purpose of finding optimal settings.  Table 6 gives the levels of experimental factors. The factors were varied at three levels: high, central, and low.

The BBD is not directly based on a full factorial design, as it uses middle points instead of corner points. The number of experiments (*n*) required for the BBD is computed by using the following equation:

(2) $$n=2k\left(k-1\right)+C_{o},$$

where *k* is the number of factors and  *C*_*o*_  is the number of central points.  Table 7 shows the mean results of 15 trials with two replicates (*k* = 3, *C*_*o*_ = 3). 

Estimated regression coefficients and related statistical terms are shown in Table 8. The effects are statistically significant when *P* value, defined as the smallest level of significance leading to rejection of null hypothesis, is less than 0.05.

Based on Table 8, the terms, which seem insignificant compared to other effects, were neglected one by one, and the related statistics were then recalculated with remaining variables.  Table 9 shows the results of the reduced model.

The ANOVA results in Table 10 indicate the relative importance of the first-order and second-order sources with respect to the sum of squares. The order of factors from high to low contribution on concentration of leached copper is stirring time, temperature∗temperature, pH, and temperature.

**Figure 1:**
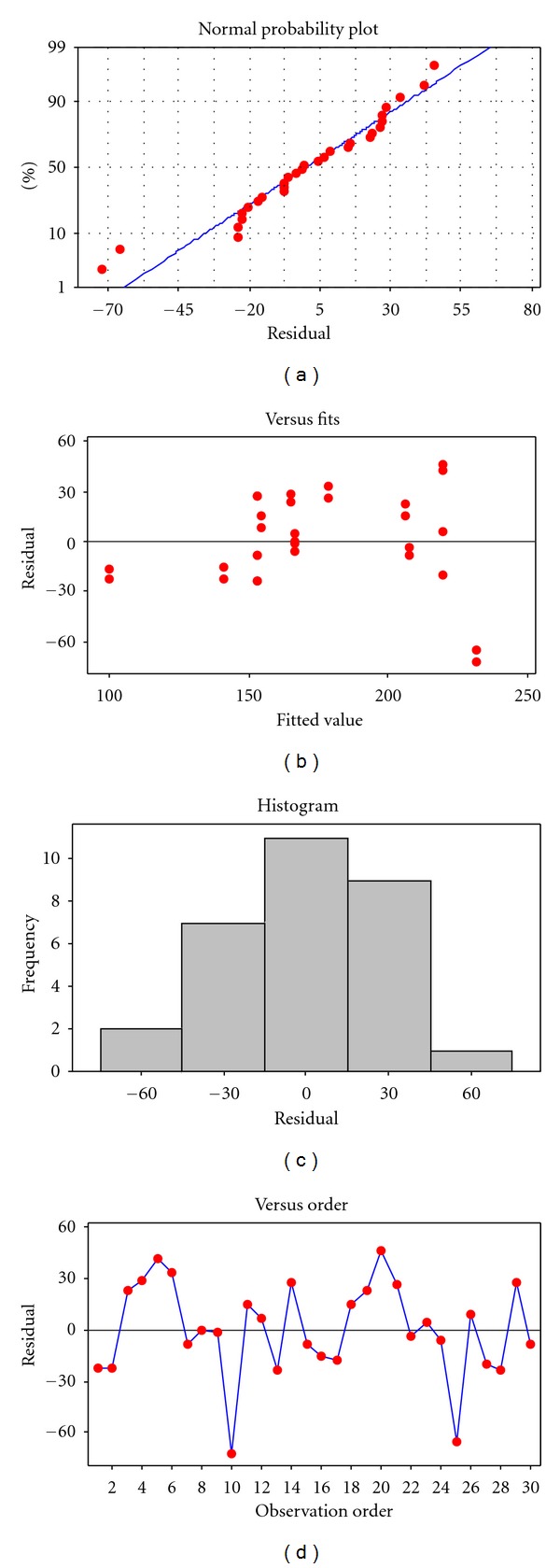
Residual plots for concentration of leached copper.

The design of experimental analysis assumes that the residuals are normally and independently distributed with the same variance in each treatment or factor level. Departures from this assumption mean that the residuals contain structure that is not accounted for in the model. Residual plots given in Figure 1 were used to check the assumption. Since the residuals lie approximately along a straight line and any pattern, such as sequences of positive and negative residuals, is not observed, it was concluded that the residuals are normally and independently distributed.

*R* Square given in Table 8 means that 55.53% of the total variation in the concentration of leached copper from copper flotation waste can be attributed to the studied experimental factors. This value is lower than expected. That is, there are other experimental factors, such as liquid/solid ratio and particle size that need to be considered. Based on the coefficients given in Table 8, the second-order prediction equation can be written as follows:

(3) $$\begin{array}{cc} y & =331.331-6.835\cdot \text{pH}+0.449\cdot \text{ST}\\ & \;-8.295\cdot T+0.100\cdot T^{2}, \end{array}$$

where *y* = concentration of leached copper (mg/L), ST = stirring time (min.), *T* = temperature (°C).

According to (3), only temperature has the second-order term. Thus, optimum value for temperature can be found as follows:

(4) $$\frac{\partial y}{\partial T}=-8.295+0.2\cdot T=0\Rightarrow T_{\text{opt}}=41.475^{{}^{\circ}}\text{C}.$$

**Figure 2:**
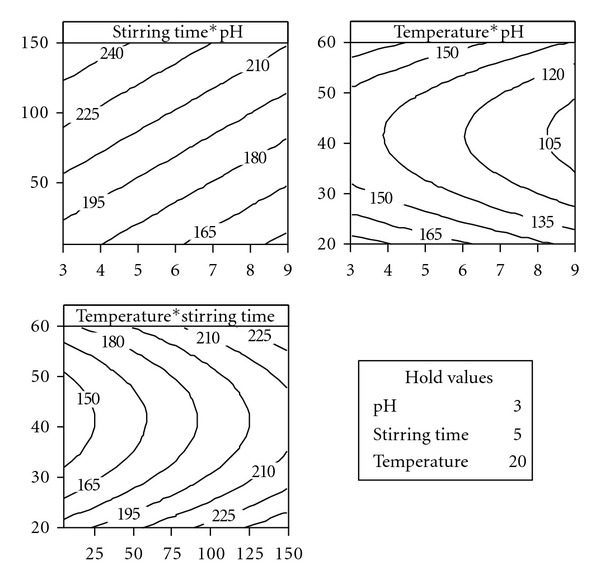
Contour plots of concentration of leached copper.

A contour plot provides a two-dimensional view where all points that have the same response are connected to produce contour lines of constant responses.  Figure 2 shows the relationship between concentration of leached copper and experimental variables. The variables not displayed in the graphs were held constant at low levels. From these figures, it is possible to determine the values of experimental factors for a specified concentration. For example, for a concentration of leached copper lower than 105 mg/L, it is necessary to set pH at 8.5–9 and temperature at 35–45°C, while stirring time is held constant at 5 minutes.

**Figure 3:**
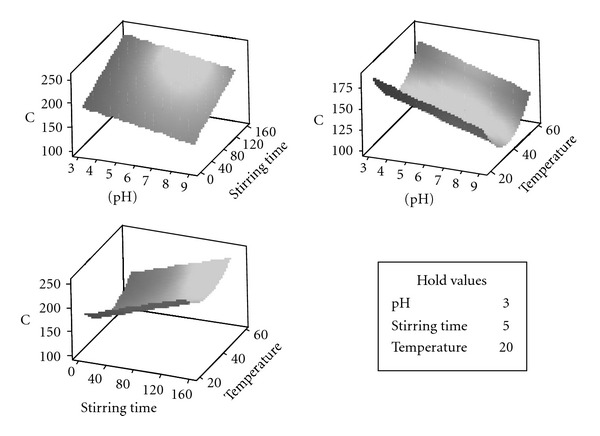
Surface plots of concentration of leached copper.

The surface plot is used to see a graphic representation of how two factors at one time affect the output (concentration) together. Since there are more than two factors, the factors not displayed in graphs are held constant. From Figure 3, it is observed that surface plots, including temperature, have a concavity characteristic due to optimum value. The lowest concentrations were found with pH at 9, stirring time at 5 minutes, and temperature at 41.5°C.

## 4. Conclusion

The copper flotation waste generated from the copper industry is classified as “hazardous waste” according to literature. In this study, a series of experiments varying the pH, stirring time, and temperature were performed to study heavy metal release from copper flotation waste. Despite the fact that leaching studies have been extensively used for the waste material, the leaching characteristics of the copper flotation waste have not been investigated before by using experimental design approach in literature. This study has given a regression equation for prediction and showed the effects of experimental factors and their interactions on concentration of leached copper.

According to the experimental results and statistical analysis, heavy metal release is strongly influenced by the pH. Under the experimental condition of this study, the solution of prediction equation for pH = 9, ST = 5 min, and *T* = 41.5°C gives the minimum concentration of leached copper, which is 100 mg/L. This value is still considerably higher than the allowed Turkish limit (Cu: 3 mg/L). Because of this, it is suggested to apply immobilization methods to decrease the concentration.

## Figures and Tables

**Table 1:** Chemical composition (wt. %) of copper flotation waste.

	Copper flotation waste
SiO_2_	24.87
Fe_2_O_3_	67.68
Al_2_O_3 _	0.92
TiO_2 _	0.08
CaO	0.69
CuO	0.98
ZnO	2.78
PbO	0.21
Cr_2_O_3_	0.12
SO_3_	2.18
K_2_O	0.48
MgO	0.36
BaO	0.10
CoO	0.21
MnO	0.12

**Table 2:** The levels of experimental factors for the full factorial design.

Factors	Symbols	Low level (−1)	High level (+1)
pH	*X*_1_	3	9
Stirring time (minutes)	*X*_2_	5	150
Temperature (°C)	*X*_3_	20	60

**Table 3:** Design matrix of the 2^3^ full factorial design.

Run no.	pH	Stirring time (min)	Temperature (°C)	Average concentration of leached copper (mg/L)
1	3 (−1)	5 (−1)	20 (−1)	221.00
2	9 (+1)	5 (−1)	20 (−1)	146.95
3	3 (−1)	150 (+1)	20 (−1)	209.90
4	9 (+1)	150 (+1)	20 (−1)	132.20
5	3 (−1)	5 (−1)	60 (+1)	191.00
6	9 (+1)	5 (−1)	60 (+1)	133.70
7	3 (−1)	150 (+1)	60 (+1)	228.75
8	9 (+1)	150 (+1)	60 (+1)	216.4
9	6 (0)	77.5 (0)	40 (0)	129.00
10	6 (0)	77.5 (0)	40 (0)	180.00
11	6 (0)	77.5 (0)	40 (0)	145.00

**Table 4:** Analysis of variance for concentration of leached copper.

Source	DF	Seq SS	Adj SS	Adj MS	*F*	*P*
Main effects	3	15385.8	15385.8	5128.6	33.71	0.000
pH	1	12254.5	12254.5	12254.5	80.54	0.000
Stirring time	1	2237.3	2237.3	2237.3	14.70	0.003
Temperature	1	894.0	894.0	894.0	5.88	0.036

(2) Way interactions	3	7462.4	7462.4	2487.5	16.35	0.000
pH∗stirring time	1	426.4	426.4	426.4	2.80	0.125
pH∗temperature	1	1685.1	1685.1	1685.1	11.07	0.008
Stirring time∗temperature	1	5350.9	5350.9	5350.9	35.17	0.000

(3) Way interactions	1	590.5	590.5	590.5	3.88	0.077
pH∗stirring time∗temperature	1	590.5	590.5	590.5	3.88	0.077
Curvature	1	2861.3	2861.3	2861.3	18.81	0.001

Residual error	10	1521.6	1521.6	152.2		
Pure error	10	1521.6	1521.6	152.2		

Total	18	27821.6				

**Table 5:** Estimated effects and coefficients for concentration of leached copper.

Term	Effect	Coef.	SE Coef.	*T*	*P*
Constant		184.99	3.084	59.99	0.000
pH	−55.35	−27.67	3.084	−8.97	0.000
Stirring time	23.65	11.83	3.084	3.83	0.003
Temperature	14.95	7.47	3.084	2.42	0.036
pH∗stirring time	10.33	5.16	3.084	1.67	0.125
pH∗temperature	20.52	10.26	3.084	3.33	0.008
Stirring time∗temperature	36.57	18.29	3.084	5.93	0.000
pH∗stirring time∗temperature	12.15	6.07	3.084	1.97	0.077
Ct Pt		−33.65	7.761	−4.34	0.001

*S* = 12.3351, PRESS = 6057.65, *R*-Sq = 94.53%, *R*-Sq (pred) = 78.23%, and *R*-Sq (adj) = 90.16%.

**Table 6:** The levels of experimental factors for the Box-Behnken experimental design.

Factors	Symbols	Low level (−1)	Center Level (0)	High level (+1)
pH	*X*_1_	3	6	9
Stirring time (minutes)	*X*_2_	5	77.5	150
Temperature (°C)	*X*_3_	20	40	60

**Table 7:** Design matrix of the Box-Behnken design.

Run no.	pH	Stirring time (min)	Temperature (°C)	Concentration of leached copper (mg/L)
1	3 (−1)	5 (−1)	40 (0)	121.80
2	9 (+1)	5 (−1)	40 (0)	79.90
3	3 (−1)	150 (+1)	40 (0)	224.85
4	9 (+1)	150 (+1)	40 (0)	191.00
5	3 (−1)	77.5 (0)	20 (−1)	213.30
6	9 (+1)	77.5 (0)	20 (−1)	208.40
7	3 (−1)	77.5 (0)	60 (+1)	201.40
8	9 (+1)	77.5 (0)	60 (+1)	168.00
9	6 (0)	5 (−1)	20 (−1)	162.45
10	6 (0)	150 (+1)	20 (−1)	162.50
11	6 (0)	5 (−1)	60 (+1)	165.90
12	6 (0)	150 (+1)	60 (+1)	212.20
13	6 (0)	77.5 (0)	40 (0)	129.00
14	6 (0)	77.5 (0)	40 (0)	180.00
15	6 (0)	77.5 (0)	40 (0)	145.00

**Table 8:** Estimated regression coefficients for concentration of leached copper.

Term	Coef.	SE Coef.	*T*	*P*
Constant	438.141	77.8093	5.631	0.000
pH	−36.180	16.1679	−2.238	0.037
Stirring time	0.538	0.5119	1.052	0.305
Temperature	−9.494	2.4252	−3.915	0.001
pH∗pH	2.087	1.1702	1.783	0.090
Stirring time∗stirring time	−0.003	0.0020	−1.493	0.151
Temperature∗temperature	0.100	0.0263	3.813	0.001
pH∗stirring time	0.009	0.0465	0.199	0.844
pH∗temperature	0.090	0.1686	0.531	0.601
Stirring time∗temperature	0.008	0.0070	1.143	0.267

*S* = 28.6208, PRESS = 38892.6, *R*-Sq = 72.10%, *R*-Sq (pred) = 33.77%, and *R*-Sq (adj) = 59.55%.

**Table 9:** Estimated regression coefficients for concentration of leached copper (reduced).

Term	Coef.	SE Coef.	*T*	*P*
Constant	331.331	44.6537	7.420	0.000
pH	−6.835	2.5007	−2.733	0.011
Stirring time (ST)	0.449	0.1035	4.340	0.000
Temperature (*T*)	−8.295	2.2282	−3.723	0.001
Temperature∗temperature (*T* ^2^)	0.100	0.0275	3.637	0.001

*S* = 30.0083, PRESS = 33253.1, *R*-Sq = 61.66%, *R*-Sq (pred) = 43.37%, and *R*-Sq (adj) = 55.53%.

**Table 10:** Analysis of variance for concentration of leached copper.

Source	DF	Seq SS	Adj SS	Adj MS	*F*	*P*
Regression	4	36208.1	36208	9052.0	10.05	0.000
Linear	3	24297.1	36174	12057.9	13.39	0.000
pH	1	6728.1	6728	6728.1	7.47	0.011
Stirring time	1	16965.1	16965	16965.1	18.84	0.000
Temperature	1	603.9	12481	12480.7	13.86	0.001
Square	1	11911.0	11911	11911.0	13.23	0.001
Temperature∗temperature	1	11911.0	11911	11911.0	13.23	0.001

Residual error	25	22512.5	22513	900.5		
Lack-of-fit	8	19249.3	19249	2406.2	12.53	0.000
Pure error	17	3263.3	3263	192.0		

Total	29	58720.6				
